# Telomere shortening and accelerated aging in COPD: findings from the BODE cohort

**DOI:** 10.1186/s12931-017-0547-4

**Published:** 2017-04-13

**Authors:** Córdoba-Lanús Elizabeth, Cazorla-Rivero Sara, Espinoza-Jiménez Adriana, Juan P. de-Torres, Pajares María-José, Aguirre-Jaime Armando, Celli Bartolomé, Casanova Ciro

**Affiliations:** 1 Research Unit, Hospital Universitario Nuestra Señora de Candelaria, Ctra. del Rosario 145, 38010 Santa Cruz de Tenerife, Spain; 2 Pulmonary Division, Hospital Universitario Nuestra Señora de Candelaria, Santa Cruz de Tenerife, Spain; 3 Pulmonary Division, Clínica Universitaria de Navarra, Pamplona, Spain; 4 Centro de Investigación Médica Aplicada (CIMA), UNAV, Pamplona, Spain; 5 Pulmonary and Critical Care Department, Brigham and Women’s Hospital, Boston, MA USA

**Keywords:** COPD, Telomeres, Aging

## Abstract

**Background:**

Chronic Obstructive Pulmonary Disease (COPD) may be associated with accelerated aging. Telomere shortening is a biomarker of aging. Cross-sectional studies describe shorter telomeres in COPD compared with matched controls. No studies have described telomere length trajectory and its relationship with COPD progression. We investigated telomere shortening over time and its relationship to clinical and lung function parameters in a COPD cohort and smoker controls without COPD.

**Methods:**

At baseline leukocyte telomere length was measured by qPCR in 121 smokers with COPD and 121 without COPD matched by age (T/S_0_). The measurements were repeated in 70 of those patients with COPD and 73 non-COPD smokers after 3 years of follow up (T/S_3_).

**Results:**

At initial measurement, telomeres were shorter in COPD patients when compared to smoker controls (T/S = 0.68 ± 0.25 vs. 0.88 ± 0.52, *p* = 0.003) independent from age and sex. During the follow-up period, we observed an accelerated telomere shortening in individuals with COPD in contrast to smoker controls (T/S_0_ = 0.66 ± 0.21 vs. T/S_3_ = 0.46 ± 0.16, *p* < 0.001, for the patients with COPD and T/S_0_ = 0.83 ± 0.56 vs. T/S_3_ = 0.74 ± 0.52, *p* = 0.023 for controls; GLIM, *p* = 0.001). This shortening was inversely related to the baseline telomere length (*r* = −0.49, *p* < 0.001). No significant relationship was found between the rate of change in telomere length and change in lung function in the patients with COPD (*p* > 0.05).

**Conclusions:**

Compared with smokers, patients with COPD have accelerated telomere shortening and this rate of attrition depends on baseline telomere length. Furthermore, the telomere length and its rate of shortening did not relate to clinical and lung function parameters changes over 3 years of follow-up.

**Electronic supplementary material:**

The online version of this article (doi:10.1186/s12931-017-0547-4) contains supplementary material, which is available to authorized users.

## Background

Chronic obstructive pulmonary disease (COPD), a major cause of morbidity and mortality throughout the world, is thought to result from the interaction of environmental agents such as tobacco smoking or exposure to biomass fuel and inherited genetic factors [1]. COPD is a multidimensional disease that frequently coexists with other age-related co-morbidities such as osteoporosis, cardiovascular disease, lung cancer, depression and diabetes [2, 3].

It has been suggested that COPD is a disease of accelerated aging [4, 5] and telomere length has been proposed as a biomarker of aging [6, 7]. Telomeres consist of stretches of repetitive hexanucleotides (5´-TTAGGG-3´) that protect the end of chromosomes from being recognized as double-strand breaks and avoid truncation. Because the DNA cannot be duplicated at the end of the chromosome, each duplication results in its shortening. Thus, telomeres get progressively shorter as cells divide, an event that is known as the end-replication process. Other mechanisms may also account for the accelerated loss of telomeres, such as the DNA damage induced by oxidative stress [8].

There is no uniform acceptance of the interaction between telomere length and COPD. However, some studies have shown that patients with COPD exhibit shorter telomeres in circulating leukocytes compared to age matched smokers without COPD [9, 10]. Some authors have proposed that telomere shortening might be accelerated in patients with COPD and serve as a biomarker of disease progression [11], a theory that has some clinical support because the telomere length in peripheral leukocytes of patients with COPD has been related to important health outcomes such as all-cause and cancer mortality [12].

The unanswered question fueling the debate about the meaning of telomere shortening in patients with COPD is that all of the studies performed have been cross-sectional in design, not informing whether there are changes in length over time and whether there is any relationship between those changes and outcomes. That this is possible in COPD is highlighted by a recent study that analyzed leukocyte telomere trajectory in a cohort of individuals with coronary artery disease and found that this is powerfully influenced by baseline telomere length in a pattern suggestive of negative feedback regulation [13].

The aim of the present study was to investigate if telomere shortening occurs over time in patients with COPD and if this shortening is related to clinical and lung function parameters.

## Methods

### Subjects

**Fig. 1:**
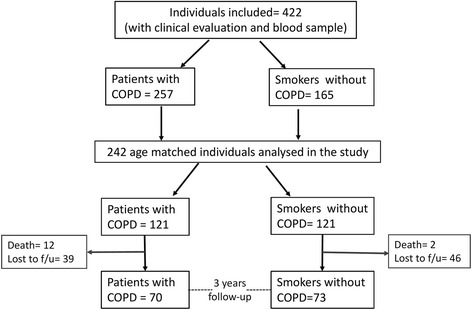
Consort diagram illustrating the selection and assessment of patients and control smokers included in the study

A total of 422 subjects were screened for this study at the Hospital Universitario La Candelaria, Tenerife and the Clinica Universitaria de Navarra, Pamplona, Spain. From these, the control group of 165 smokers without COPD had a smoking history of > 15 pack-years and normal lung function (FEV1 > 80%; FEV1/FVC ≥ 0.70). There were 257 smokers with COPD followed annually as part of the BODE study [14]. Inclusion criteria: age > 40 years, smoking history >15 pack-years and post-bronchodilator FEV_1_/FVC ratio < 0.70 measured 20 min after administration of 400 mg of albuterol. Pulmonary function test, spirometry lung volumes and exercise capacity were measured according to ATS-ERS guidelines [15–17] using the European Community for Steel and Coal as reference values [18]. Dyspnea was evaluated by mMRC scale [19]. The BODE Index was calculated as previously described [14]. Co-morbidity was quantified using the Charlson index [20]. Patients were clinically stable (no exacerbation for at least 6 weeks) at the time of evaluation. Repeated determinations of the complete blood count revealed similar values at all visits. Exclusion criteria: uncontrolled co-morbidities such as malignancy at baseline, asthma or other pulmonary conditions than COPD. From the screened group, 121 patients with COPD were matched with 121 smokers without COPD and were included in the present study for baseline comparison (Fig. 1). From these subjects, 70 patients with COPD age-matched with 73 control smokers returned for all follow-up evaluations over 3-years and were analyzed in the longitudinal study. The individuals included in the final analysis did not have any relevant clinical difference with the ones that did not complete the 3-year study (see Additional files 1 and 2). The study was approved by the institutional review board at both hospitals. All participants provided written informed consent.

### Telomere length measurement

Venous blood was obtained at baseline and at the third-year post enrollment. DNA was extracted from leukocytes using the QIAamp DNA Mini Kit (GE Healthcare). Telomere length was measured using a qPCR based protocol previously published [21, 22]. Briefly, DNA samples were quantified using the Nanodrop lite spectrophotometer (Thermo Scientific, Wilmington, DE, USA). Telomere length measurement was performed in triplicate using 20 ng of DNA. Intra-plate coefficients of variance (CV) were calculated between the replicates and samples with CV > 5% were excluded from further analysis. As a reference DNA sample with shorter telomeres we used the DNA from MCF-7 cells. For longer telomeres, we assayed a DNA from a young control individual. These calibrator samples were assayed in triplicate on each PCR plate to control for variation between plates. Inter-assay variability was controlled by measuring two control DNA samples per run as a normalizing factor. Inter-plate CV for the calibrator sample was calculated to be <9%. Standard curves were derived from serially diluted reference DNA. Albumin, being a single copy gene, was used as a reference gene. The primer sequences and cycling conditions for the measurement of telomere length were the same used by others [22]. qPCR reactions were performed in 20 μL reactions for each individual: 10 μL SYBRGreen PCR Master Mix (BioRad), 0.9 μM of Telg and Telc and 0.6 μM of Albu and Albd. All the reactions were performed on triplicates of the telomere and reference gene assays on the iQ Cycler Real-Time PCR Instrument (BioRad).

Telomere length was calculated as a ratio of telomere to albumin as previously described [22]. The T/S ratio for an experimental DNA sample is T, the number of nanograms of the Standard DNA that matches the experimental sample for copy number of the telomere template, divided by S, the number of nanograms of the standard DNA that matches the experimental sample for copy number of the albumin single copy gene. The telomere length standardized to the reference single copy gene (T/S) was calculated using the “∆∆Cp with efficiency correction” calculation method [23].

### Statistical analysis

For the cross-sectional analysis, patients with COPD were categorized in three groups by telomere length ratio (T/S) tertiles at baseline: shorter, medium or longer telomeres.

For the longitudinal analysis, telomere length changes were calculated as the difference between baseline (T/S_0_) and the third year (T/S_3_) of the telomere length measure. Individuals analyzed for follow-up were categorized as follows: subjects showing a steeper rate of telomere shortening in contrast to subjects with a lower telomere shortening rate, maintenance or lengthen of their telomeres (defined by the median telomere length change).

A t-Student, ANOVA, Chi^2^ and Paired Sample *T* test were used to test differences in means and proportions of baseline and follow-up characteristics between groups. The association between baseline telomere length (T/S) or telomere length change with clinical and/or pulmonary function variables was explored using Pearson’s correlation coefficients. A multiple logistic regression was performed to test the association of telomere length with COPD susceptibility adjusting for age, sex and pack years. By using a general linear modelling for repeated measures (GLIM) we tested the telomere dynamics difference between patients with COPD and smokers without COPD, or within these groups between current and former smokers. We also conducted a longitudinal analysis to evaluate the progression of the disease of clinical and pulmonary function variables using change in telomere length as comparative factor (GLIM). SPSS 20.0 IBM Co software was used for all statistical analyses and two-tailed *p*-values < 0.05 were considered significant.

## Results

### Baseline findings

The clinical characteristics and lung function data of the 121 COPD patients and 121 controls at baseline, are summarized in Table 1. The range of airflow obstruction distributed by GOLD stages in COPD was as follows: 1 (19.8%), 2 (43%), 3 (28.9%) and 4 (8.3%). As expected, patients with COPD had worse lung function and a higher BODE index than controls. However, the two groups were similar in age, sex, BMI and Charlson co-morbidity score. Although the total pack years smoking was higher in COPD patients, there were more current smokers in the control group. Telomere length measured by the T/S ratio inversely correlated with age in COPD (*r* = −0.21; *p* = 0.02) and in smoker controls (*r* = −0.19; *p* = 0.02). Given this relationship, all subsequent analyses were adjusted by age.

**Table 1:** Baseline characteristics of COPD patients and smokers without COPD included in the study

Variable	COPD cases (*N* = 121)	Smokers (*N* = 121)	*p*-value
Sex (male%)	66	62	N.S.
Age (years)^a^	57 ± 8	57 ± 8	N.S.
BMI (Kg/m^2^)^a^	27 ± 5	28 ± 4	N.S.
Smoking habit^c^ (pack-yrs)^ac^	60 ± 25	44 ± 22	<0.0001
Active smoking (%)	50	64	0.027
T/S ratio^a^	0.68 ± 0.25	0.88 ± 0.52	<0.0001
FEV_1_ (L)^a^	1.66 ± 0.7	2.9 ± 0.7	<0.0001
FEV_1_ (% pred)^a^	59 ± 21	101 ± 14	<0.0001
FVC (% pred)^a^	89 ± 22	108 ± 15	<0.0001
FEV_1_ / FVC (%)^a^	53 ± 12	76 ± 5	<0.0001
PaO_2_ (mmHg)^a^	72.3 ± 14.3	78.7 ± 10	<0.001
K_CO_ (%)	79.6 ± 25.1	97.3 ± 21.3	0.001
IC/TLC (%)	36 ± 9	45 ± 8	<0.001
6MWD (m)^a^	523 ± 89	551 ± 76	0.03
mMRC dyspnea^b^	1 (0–2)	0 (0–0)	0.006
BODE index^b^	1 (0–3)	0 (0–1)	<0.001
Charlson index^b^	0 (0–1)	0 (0–0)	N.S.

Data are presented as ^a^mean ± SD and ^b^median (25^th^-75^th^pc) and compared by Student’s *t*-test or Chi_2_ test. ^c^Number of packs of cigarettes smoked per day x number of years smoking. Percentage *BMI* body mass index, *T/S ratio* relative telomere length, *FEV*_*1*_ forced expiratory volume in one second, *FVC* forced vital capacity, *% pred* per cent predicted. *PaO*_*2*_ partial oxygen tension, *K*_*CO*_ diffusion capacity of carbon monoxide, *IC/TLC* inspiratory to total lung capacity ratio, *6MWD* six-min walk distance test. *N.S* non-significant

**Fig. 2:**
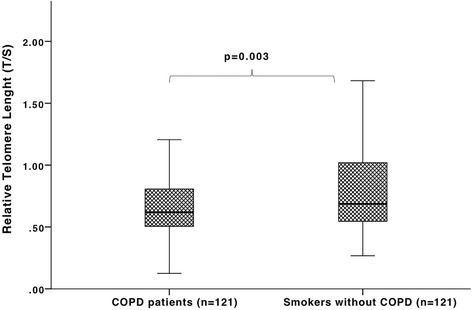
Average telomere length and SE relative telomere length (T/S) in COPD patients (*n* = 121) vs. age-matched smokers without COPD (*n* = 121)

COPD patients had significant shorter telomeres than smokers without COPD (T/S = 0.68 ± 0.25 vs. 0.88 ± 0.52, *p* < 0.0001) even after adjusting for age, sex and pack-years (95% CI: 0.11–0.64, *p* = 0.003), (Table 1 and Fig. 2). There were no significant differences between males (0.68 ± 0.28) and females (0.66 ± 0.20) within the COPD group or in the smokers (0.82 ± 0.53 vs. 0.96 ± 0.49 for males and females, respectively).

There was no relationship between telomere length and the clinical and lung function parameters (Table 2) or between patients with less and more severe COPD (GOLD 1–2 vs. GOLD 3–4 or BODE ≤2 vs. BODE > 2) (*p* > 0.05). In addition, no significant associations were found between comorbidities such as cardiovascular events, diabetes, hypertension or cancer and relative telomere length in patients with COPD.

**Table 2:** Pulmonary function characteristics of the three groups of patients with COPD defined by their baseline telomere length

Variable	Short T/S^a^ (*n* = 40)	Medium T/S^a^ (*n* = 41)	Long T/S^a^ (*n* = 40)	*p*-value
T/S ratio^b^	0.44 ± 0.09	0.66 ± 0.07	1.09 ± 0.21	<0.001
Sex (male%)	65	66	67	N.S.
Age (years)^b^	59 ± 7	58 ± 8	55 ± 9	N.S.
BMI (Kg/m^2^)^b^	28 ± 6	27 ± 5	27 ± 5	N.S.
Smoking habit^d^ (pack-yrs)^bd^	62 ± 28	63 ± 24	57 ± 23	N.S.
Active smoking (%)	42	56	52	N.S.
FEV_1_ (L)^b^	1.70 ± 0.71	1.64 ± 0.67	1.66 ± 0.74	N.S.
FEV_1_ (% pred)^b^	61 ± 23	59 ± 21	58 ± 21	N.S.
FVC (% pred)^b^	89 ± 24	92 ± 22	87 ± 21	N.S.
FEV_1_ / FVC (%)^b^	55 ± 12	51 ± 13	53 ± 12	N.S.
PaO_2_ (mmHg)^b^	71.1 ± 15.7	71.9 ± 15.8	73.5 ± 11.5	N.S.
K_co_ (%)	74.1 ± 25.6	75.5 ± 24.1	75.2 ± 21.1	N.S.
IC/TLC (%)	37 ± 6	36 ± 8	35 ± 10	N.S.
6MWD (m)^b^	517 ± 77	537 ± 95	513 ± 92	N.S.
mMRC dyspnea^c^	1 (0–2)	1 (0–2)	1 (0–2)	N.S.
BODE index^c^	1 (0–2)	1 (0–3)	1 (0–3)	N.S.
Charlson index^c^	0 (0–1)	0 (0–1)	0 (0–1)	N.S.

^a^Groups defined by telomere length (T/S) tertiles: <0.54, 0.54–0.73 and >0.73. Data are presented as ^b^mean ± SD and ^c^median (25^th^-75^th^pc) and compared by ANOVA test or Chi_2_ test. ^d^Number of packs of cigarettes smoked per day x number of years smoking. *BMI* body mass index, *T/S ratio* relative telomere length, *FEV*_*1*_ forced expiratory volume in one second, *FVC* forced vital capacity, *% pred* per cent predicted. *PaO*_*2*_ partial oxygen tension, *K*_*CO*_ diffusion capacity of carbon monoxide, *IC/TLC* inspiratory to total lung capacity ratio, *6MWD* six-min walk distance test. *N.S* non-significant

### Telomere length dynamics

This difference in telomere length remained significant after 3 years between the 70 patients with COPD age matched with 73 smoker controls that presented the follow-up (T/S_3_ = 0.46 ± 0.16 vs. 0.74 ± 0.52, *p* < 0.001); even after adjusting for age, sex and smoking habit in a logistic regression analysis (95% CI: 0.12–0.90), *p* = 0.03).

**Fig. 3:**
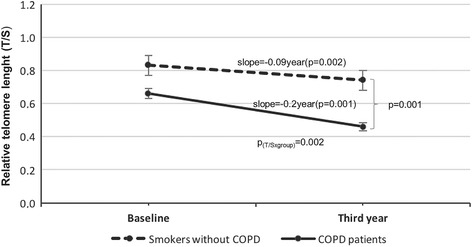
Telomere length (relative T/S ratio) dynamics in COPD patients (*n* = 70) vs. age-matched smokers without COPD (*n* = 73) after 3 years’ follow-up (*p* = 0.001, using GLIM - general lineal model for repetitive measures; T/S differences between groups p_(T/Sxgroups)=_0.002)

The telomere length ratio in the control group of smokers without COPD, decreased significantly (T/S_0_ = 0.83 ± 0.56 vs. T/S_3_ = 0.74 ± 0.52, *p* = 0.002) after the follow-up period. The same occurred in the group of patients with COPD (T/S_0_ = 0.66 ± 0.21 vs. T/S_3_ = 0.46 ± 0.16, *p* < 0.001). However, when we compared the telomere trajectory between these groups over the follow-up period, we observed an accelerated telomere shortening in individuals with COPD in contrast to the control group (GLIM, general lineal model for repetitive measures, *p* = 0.001) (Fig. 3).

### Relation of telomere length change and clinical variables

**Fig. 4:**
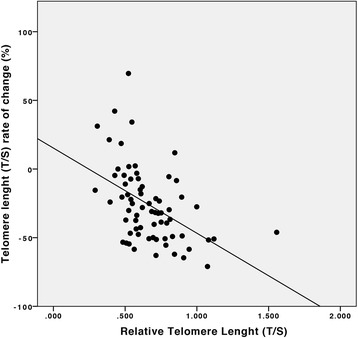
Correlation between baseline telomere length and the rate of change in telomere shortening in COPD patients after 3 years’ follow-up (*r* = −0.49, *p* = 0.001, adjusted by age)

We found a strong relationship between baseline telomere length and change in telomere length (*r* = −0.49, *p* < 0.001; adjusted by age) (Fig. 4).

Compared with COPD patients that did not change, lengthened or had a low rate of telomere shortening, those patients with a more rapid shortening of their telomeres had similar lung function characteristics (Table 3).

**Table 3:** Pulmonary function variables over time and telomere length rate of change in COPD patients

Variable	High rate of shortening^b^ (*n* = 35)			Low rate of shortening maintenance or lengthening^c^ (*n* = 35)		
	Baseline	3-years	*p*-value	Baseline	3-years	*p*-value
FEV_1_ (L)	1.66 ± 0.72	1.59 ± 0.68	0.04	1.60 ± 0.5	1.52 ± 0.5	0.15
FEV_1_(%pred)	59 ± 20	58 ± 20	0.63	59 ± 19	58 ± 19	0.54
FVC (L)	3.01 ± 0.99	2.95 ± 0.98	0.35	3.15 ± 0.94	3.01 ± 0.97	0.12
FEV_1_/FVC	54 ± 10	53 ± 11	0.21	52 ± 12	52 ± 13	0.77
PaO_2_ (mmHg)	73.5 ± 7.9	70.9 ± 9.6	0.04	72.1 ± 12.9	68.8 ± 11.6	0.09
K_CO_ (%)^a^	77.3 ± 23.7	74.9 ± 23.4	0.21	74.4 ± 19.1	70.6 ± 20.4	0.16
IC/TLC (%)	35 ± 9	31 ± 10	0.03	36 ± 8	32 ± 8	0.02

Data are presented as mean ± SD and were compared by Paired Sample *T*-Test. *FEV*_*1*_ forced expiratory volume in one second, *FVC* forced vital capacity, *% pred* per cent predicted, *PaO*_*2*_ partial oxygen tension, *K*_*CO*_ diffusion capacity of carbon monoxide, *IC/TLC* inspiratory to total lung capacity ratio. ^a^Subjects consider to analysis in each group (*n* = 17). ^b^Below the median of telomere length rate of change. ^c^Upper the median telomere length rate of change

By using correlation matrices, we were not able to find any significant relationship between the rate of change in telomere length and change in lung function in the patients with COPD (see Additional file 3). This lack of association was seen whether we compared current vs. former smokers, number of comorbidities or mortality (Chi_2_, *p* > 0.05).

## Discussion

This longitudinal study shows that COPD patients experience an accelerated telomere shortening process compared with smokers without COPD and this telomere attrition occurs in close relation to baseline telomere length. The study also confirms that patients with COPD have shorter telomeres than smokers who do not have COPD. However, there were no associations between telomere length and clinical and lung function parameters at baseline or between changes in telomere length and change in those parameters overtime. To our knowledge, this is the first study reporting the course of telomere length change in a well-characterized cohort of patients with COPD and smoker controls.

### Telomere length and change over time

In our study, at baseline, telomere length was shorter with older age, both in the COPD patients and the smokers without COPD. However, COPD patients presented shorter telomeres than the control group independent of age and sex. Other studies evaluating the relation between telomere length and COPD have shown discordant results. Morla et al. [24] failed to find a difference in telomere length between COPD patients and smokers, whereas some other studies have reported shorter telomeres in subjects with COPD compared to smokers and healthy individuals [8, 10]. These discordant results may have been due to differences in sample size in the studies or by technical bias or defects in study design, some of which lacked of appropriate controls. We found no relationship between telomere length and clinical or lung function parameters in our COPD cohort in the cross-sectional analysis. This is in agreement with the results of Savale et al. [9] who described a borderline correlation between PaO_2_ and PaCO_2_ and telomere length but strangely not to lung function parameters. However, Rode et al. [25] in a study of more than 45.000 individuals in a Danish population reported a modest correlation between telomere length and the lung function expressed by the FEV_1_. More recently, Rutten et al. studying several markers of aging in patients with COPD, found telomere length to be the only one associated with lung function [26]. All these studies were cross-sectional in design and in consequence not adequate to evaluate the possible relationship between telomere length and COPD progression. In addition, the only lung function parameter evaluated was FEV_1_ and patients with comorbidities were excluded.

The current report is the first one to document that COPD patients have an accelerated rate of telomere shortening in contrast to smoker controls, and this difference was more marked in those individuals with longer baseline telomeres. In our study, 85% of the COPD patients showed telomere length shortening after 3 years of follow-up, a proportion that is similar to that observed in general population studies [27–29], however, the rate of decline is significantly lower in those normal individuals. It is important to compare our findings with that of other populations including different pathologies. Fazarneh et al. [13] analyzed leukocyte telomere trajectory over 5 years in a cohort of individuals with coronary artery disease and found, as we have, that telomere shortening rate is powerfully influenced by baseline telomere length. Our results support a pattern suggestive of negative feedback regulation in the telomere shortening dynamics, as has been proposed by others [29]. It may be that once a critical telomere length has been reached, activation of certain mechanisms in the cells occur to prevent telomere shortening over years accounting for less attrition per replication or maintenance of viable telomere lengths in subjects with shorter telomeres.

The exact reason why telomeres shorten faster in patients with COPD was beyond the scope of this study. However, oxidative stress and inflammation are the most important factors thought to be responsible for the loss of telomeric DNA [8], which in turn may favor the development of structural tissue damage [30]. Studies on mouse models of accelerated aging and lung dysfunction, suggested that this process might enhance susceptibility to COPD [5].

### Telomere length and clinical variables

Previous cross-sectional studies have concentrated primarily on the relationship between telomere length and the FEV_1_. We expanded on those studies relating not only FEV_1_, but also lung hyperinflation (IC/TLC), diffusion capacity (K_CO_) and gas exchange to telomere length. Disappointingly, we were unable to document any association between telomere length in COPD patients with any clinical or functional expression of the disease or presence of any comorbidities at baseline. This is in general agreement with most of the published studies that have reported poor associations, if any, between the actual telomere length and the FEV_1_. Of interest, the studies that have described an association between telomere shortening and lower FEV_1_ have included larger sample size, thus facilitating a statistical significance to those findings. This is important when attempting to relate statistical associations to practical the clinical application of those findings.

Because the value of a biomarker resides primarily in its capacity to reflect association to prognosis or to change in disease progression, we followed patients and controls over 3 years. We observed no correlation between the rate of shortening of telomeres and the change in lung function, or for that matter with any other clinical variable, rendering those findings difficult to interpret of to apply clinically. Perhaps, a longer period of observation or a larger sample may be necessary to evaluate these clinical and physiological variables or other patient related outcomes such as exacerbations, cardiovascular events and all-cause mortality. On the other hand, these findings suggest that the rate of telomere shortening may not be a viable surrogate marker of disease progression of major clinical utility.

This study has several limitations. Telomere length was measured on circulating leukocytes and therefore our findings may not reflect the process occurring in the whole lung. Interestingly, telomere length in blood correlates well with telomere length in lung tissue of COPD [31] and rates of telomere shortening are similar in different tissues [32]. We used qPCR relative telomere length measures rather than absolute ones which may result in a loss of precision, however qPCR results were reported to be strongly correlated those obtained with southern blot technique [22]. Leukocyte telomere length is also considered a marker of oxidative stress and inflammation, and both contribute to COPD development. Telomere length is determined by environmental as well as genetic factors. There is a large inter-individual variability in telomere length in humans of the same age that makes difficult any cross-sectional comparisons. Our control smokers presented a shorter tobacco exposure history than the cohort of patients with COPD. However, analysis between current vs. former smokers in both cohorts did not show significantly differences. Another limitation is that the sample size or duration of observation (3 years) may not have been large or long enough to find significant associations between telomere shortening and lung function parameters. In this context, we cannot discard that our findings may be affected by regression towards the mean. Further studies with a larger follow-up period with many time points of measurements are needed to validate these findings. However, for implementation of precision medicine, biomarkers need to have clinical applicability in small numbers, if not in individuals. Lastly, we did not investigate other markers of aging; however, other authors who have done so, and have found telomere length to be the most relevant of all [26].

## Conclusions

In summary, in this longitudinal observational study we found that compared with smoker controls, an accelerated telomere shortening occurs in patients with COPD, even if they had shorter telomeres at baseline. Interestingly, the speed of shortening relates inversely to baseline telomere length. However, the telomere length and its rate of shortening did not relate to clinical and lung function parameters and their change over time, making telomere length change an unlikely useful biomarker of COPD progression.

## Additional files

Additional file 1: Baseline characteristics of COPD patients with and without three-year follow-up included in the study. (DOCX 14 kb)

Additional file 2: Baseline characteristics of smokers without COPD with and without three-year follow-up included in the study. (DOCX 75 kb)

Additional file 3: Correlations between the rate of change in telomere length and the change in pulmonary function variables in patients with COPD after three years of follow-up. (DOCX 68 kb)

## Abbreviations

6MWD: 6-min walk distance
BMI: Body mass index
COPD: Chronic obstructive pulmonary disease
FEV_1_: Forced expiratory volume in one second
FVC: Forced vital capacity
GLIM: General linear modelling for repeated measures test
GOLD: Global initiative for chronic obstructive lung disease
IC/TLC: Inspiratory to total lung capacity ratio
K_CO_: Diffusion capacity of carbon monoxide
N.S.: Non-significant
PaO_2_: Partial oxygen tension
pPCR: Quantitative real time polymerase chain reaction
SD: Standard deviation
T/S: Relative telomere length ratio
T/S_0_: Relative telomere length ratio at baseline
T/S_3_: Relative telomere length ratio at the third year of follow-up
