# Erratum to: Telomere shortening and accelerated aging in COPD: findings from the BODE cohort

**DOI:** 10.1186/s12931-017-0589-7

**Published:** 2017-06-02

**Authors:** Elizabeth Cordoba-Lanus, Sara Cazorla-Rivero, Adriana Espinoza-Jimenez, Juan P. de-Torres, María J. Pajares, Armando Aguirre-Jaime, Bartolomé Celli, Ciro Casanova

**Affiliations:** 1 Research Unit, Hospital Universitario Nuestra Señora de Candelaria, Ctra. del Rosario 145, 38010 Santa Cruz de Tenerife, Spain; 2 Pulmonary Division, Hospital Universitario Nuestra Señora de Candelaria, Santa Cruz de Tenerife, Spain; 3 Pulmonary Division, Clínica Universitaria de Navarra, Pamplona, Spain; 4 Centro de Investigación Médica Aplicada (CIMA), UNAV, Pamplona, Spain; 5 Pulmonary and Critical Care Department, Brigham and Women’s Hospital, Boston, MA USA

## Erratum

Upon publication of the original article [1], it was noticed that the author's name “Elizabeth Cordoba-Lanus ” was incorrectly given as “Córdoba-Lanús Elizabeth”, the author’s name “Sara Cazorla-Rivero” was incorrectly given as “Cazorla-Rivero Sara”, the author’s name “Adriana Espinoza-Jimenez” was incorrectly given as “Espinoza-Jiménez Adriana”, the author’s name “María J. Pajares ” was incorrectly given as “Pajares María-José”, the author’s name “Armando Aguirre-Jaime” was incorrectly given as “Aguirre-Jaime Armando”, the author’s name “, Bartolomé Celli” was incorrectly given as “Celli Bartolomé” and the author’s name “Ciro Casanova” was incorrectly given as “Casanova Ciro”.This has now been acknowledged and corrected in this erratum. The original article has been corrected.
