# Supplementary material for: Telomere shortening and accelerated aging in COPD: findings from the BODE cohort
Source: Respir Res. 2017 Apr 13;18:59. doi: 10.1186/s12931-017-0547-4 (PMC5390353; doi:10.1186/s12931-017-0547-4)
Supplement: Supplementary file 1 — Baseline characteristics of COPD patients with and without three-year follow-up included in the study. (DOCX 14 kb) [file 12931_2017_547_MOESM1_ESM.docx]

**Additional file 1.** Baseline characteristics of COPD patients with and without three-year follow-up included in the study.

| **Variable** | **COPD cases**  **with 3-year follow-up (N=70)** | **COPD cases**  **without 3-year follow-up (N=51)** | **p-value** |
| --- | --- | --- | --- |
| **T/S ratio*** | 0.76±0.32 | 0.69±0.31 | N.S. |
| **Sex (male%)** | 73 | 57 | N.S. |
| **Age (years)*** | 59±9 | 54±7 | N.S. |
| **BMI (Kg/m^2^)*** | 27±5 | 27±6 | N.S. |
| **Smoking habit**^†^  **(pack-yrs)^†^*** | 63±26 | 57±24 | N.S. |
| **Active smoking (%)** | 53 | 47 | N.S. |
| **FEV_1_ (L)*** | 1.63±0.61 | 1.72±0.81 | N.S. |
| **FEV_1_ (% pred)*** | 59±20 | 60±23 | N.S. |
| **FVC (% pred)*** | 89±23 | 90±21 | N.S. |
| **FEV_1_ / FVC (%)*** | 53±11 | 54±13 | N.S. |

*Data are presented as mean ±SD. ** Data are presented as median (25^th^-75^th^pc). ^†^Number of packs of cigarettes smoked per day x number of years smoking. BMI: body mass index; T/S ratio: relative telomere length; FEV_1_: forced expiratory volume in one second; FVC: forced vital capacity; % pred: per cent predicted. N.S: non-significant.
