# Supplementary material for: Telomere shortening and accelerated aging in COPD: findings from the BODE cohort
Source: Respir Res. 2017 Apr 13;18:59. doi: 10.1186/s12931-017-0547-4 (PMC5390353; doi:10.1186/s12931-017-0547-4)
Supplement: Supplementary file 2 — Baseline characteristics of smokers without COPD with and without three-year follow-up included in the study. (DOCX 75 kb) [file 12931_2017_547_MOESM2_ESM.docx]

**Additional file 2.** Baseline characteristics of smokers without COPD with and without three-year follow-up included in the study.

| **Variable** | **Smokers**  **with 3-year follow-up (N=73)** | **Smokers**  **without 3-year follow-up (N=48)** | **p-value** |
| --- | --- | --- | --- |
| **T/S ratio*** | 0.83±0.56 | 0.95±0.44 | N.S. |
| **Sex (male%)** | 75 | 46 | 0.004 |
| **Age (years)*** | 60±7 | 53±8 | N.S. |
| **BMI (Kg/m^2^)*** | 28±4 | 28±5 | N.S. |
| **Smoking habit**^†^  **(pack-yrs)^†^*** | 45±25 | 43±17 | N.S. |
| **Active smoking (%)** | 55 | 80 | 0.004 |
| **FEV_1_ (L)*** | 2.89±0.76 | 2.86±0.68 | N.S. |
| **FEV_1_ (% pred)*** | 100±15 | 102±13 | N.S. |
| **FVC (% pred)*** | 106±15 | 109±15 | N.S. |
| **FEV_1_ / FVC (%)*** | 75±5 | 78±4 | N.S. |

*Data are presented as mean ±SD. ** Data are presented as median (25^th^-75^th^pc). ^†^Number of packs of cigarettes smoked per day x number of years smoking. BMI: body mass index; T/S ratio: relative telomere length; FEV_1_: forced expiratory volume in one second; FVC: forced vital capacity; % pred: per cent predicted. N.S: non-significant.
