# Supplementary material for: Telomere shortening and accelerated aging in COPD: findings from the BODE cohort
Source: Respir Res. 2017 Apr 13;18:59. doi: 10.1186/s12931-017-0547-4 (PMC5390353; doi:10.1186/s12931-017-0547-4)
Supplement: Supplementary file 3 — Correlations between the rate of change in telomere length and the change in pulmonary function variables in patients with COPD after three years of follow-up. (DOCX 68 kb) [file 12931_2017_547_MOESM3_ESM.docx]

**Additional file 3.** Correlations between the rate of change in telomere length and the change in pulmonary function variables in patients with COPD after three years of follow-up.

| **Patients with COPD (n=70)** | | |
| --- | --- | --- |
| **Variable** | **Pearson´s r** | **p-value^‡^** |
| **FEV_1_ (L)** | -0.09 | 0.47 |
| **FEV_1_(%pred)** | -0.05 | 0.67 |
| **FVC (L)** | -0.16 | 0.18 |
| **FVC (%pred)** | -0.11 | 0.35 |
| **FEV_1_/FVC (%)** | 0.05 | 0.67 |
| **PaO_2_ (mmHg)** | 0.21 | 0.09 |
| **K_CO_ (%)*** | 0.11 | 0.56 |
| **IC/TLC (%)** | -0.04 | 0.75 |

Abbreviations: BMI: body mass index; FEV_1_: forced expiratory volume in one second; FVC: forced vital capacity; % pred: per cent predicted. PaO_2_: partial oxygen tension; K_CO_: diffusion capacity of carbon monoxide; IC/TLC: inspiratory to total lung capacity ratio. *Subjects considered to analysis (n=36). ^‡^Correlation analysis was adjusted by age.
